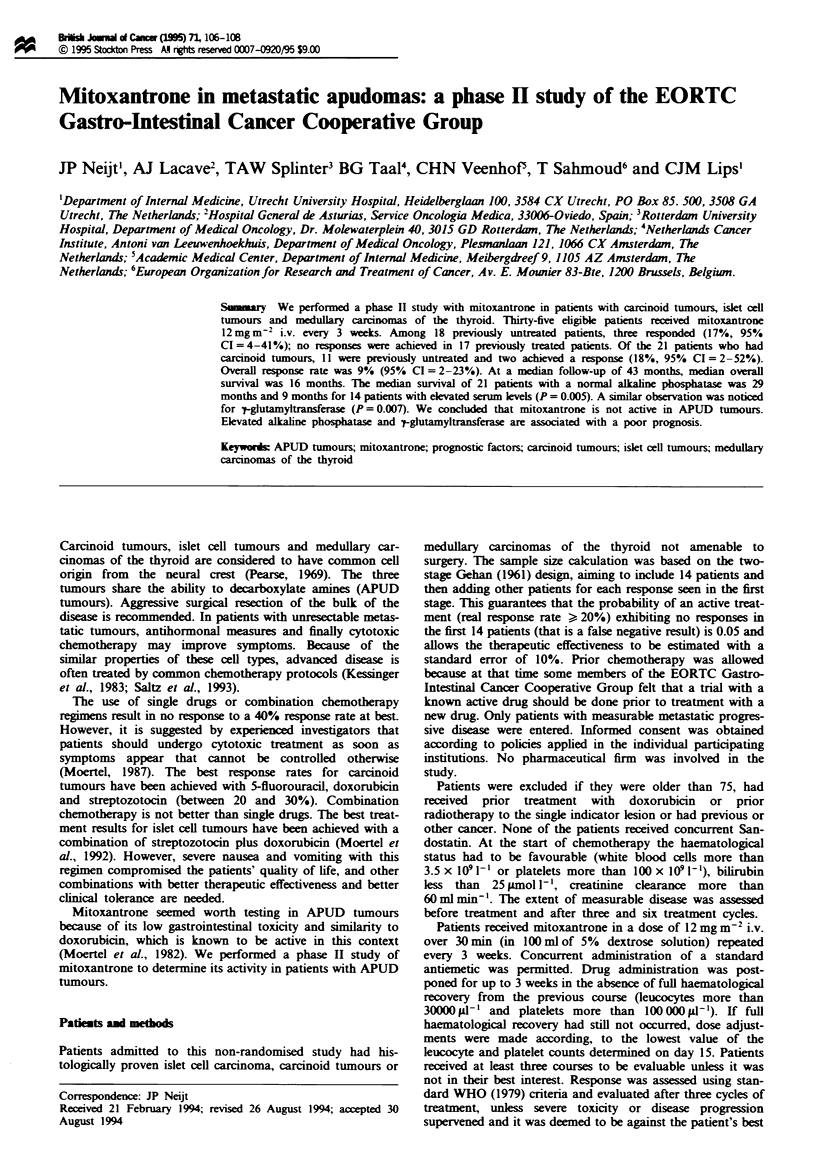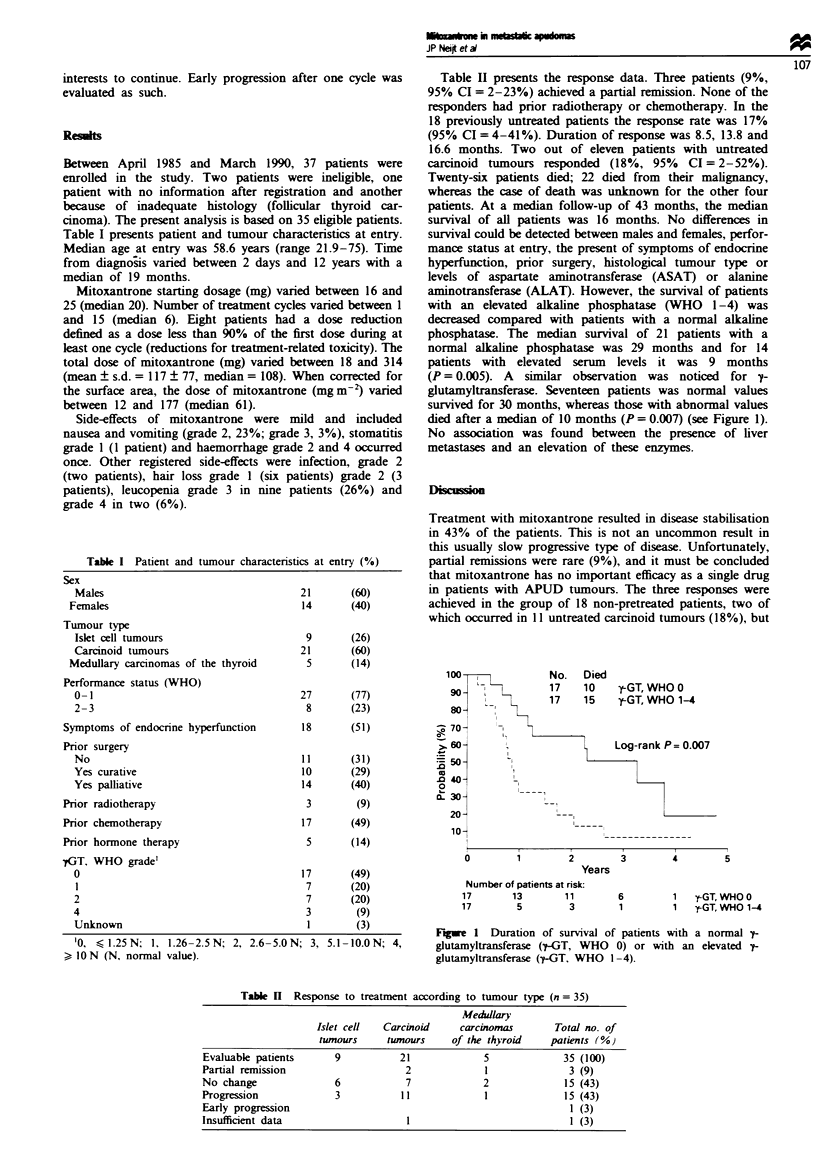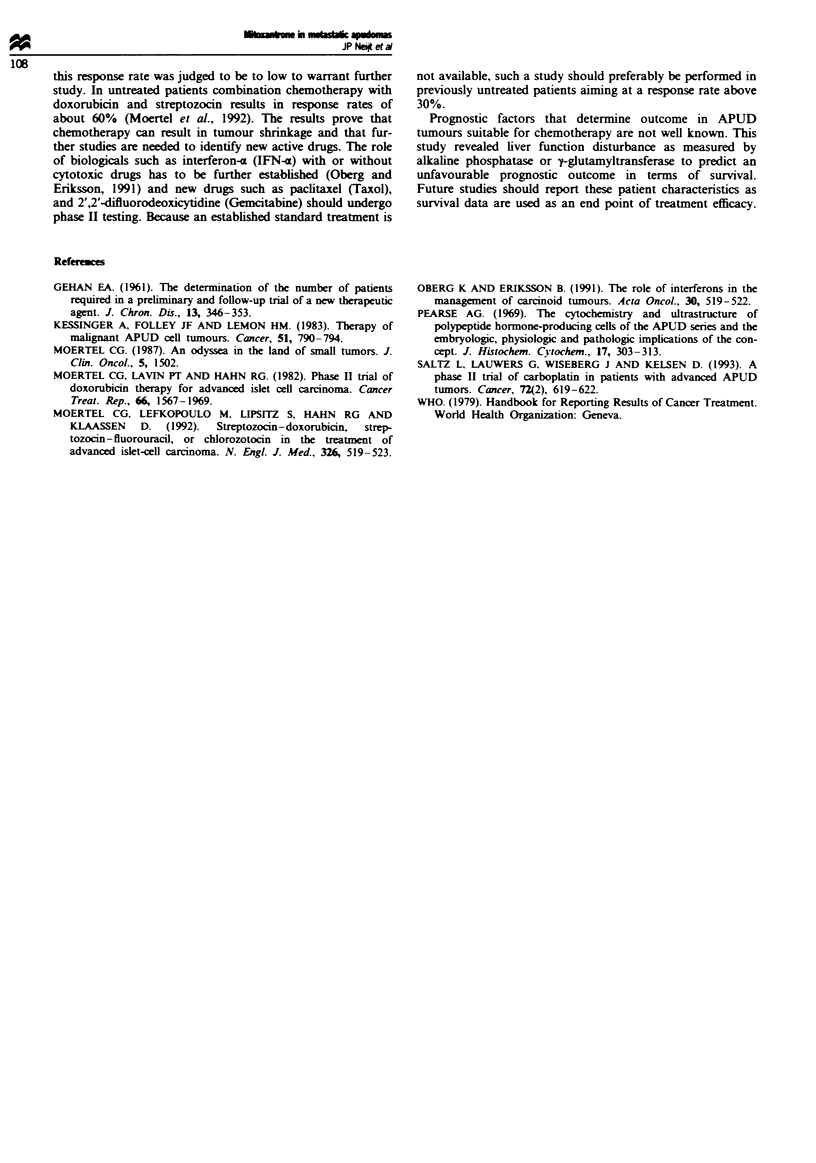# Mitoxantrone in metastatic apudomas: a phase II study of the EORTC Gastro-Intestinal Cancer Cooperative Group.

**DOI:** 10.1038/bjc.1995.21

**Published:** 1995-01

**Authors:** J. P. Neijt, A. J. Lacave, T. A. Splinter, B. G. Taal, C. H. Veenhof, T. Sahmoud, C. J. Lips

**Affiliations:** Department of Internal Medicine, Utrecht University Hospital, The Netherlands.

## Abstract

We performed a phase II study with mitoxantrone in patients with carcinoid tumours, islet cell tumours and medullary carcinomas of the thyroid. Thirty-five eligible patients received mitoxantrone 12 mg m-2 i.v. every 3 weeks. Among 18 previously untreated patients, three responded (17%, 95% CI = 4-41%); no responses were achieved in 17 previously treated patients. Of the 21 patients who had carcinoid tumours, 11 were previously untreated and two achieved a response (18%, 95% CI = 2-52%). Overall response rate was 9% (95% CI = 2-23%). At a median follow-up of 43 months, median overall survival was 16 months. The median survival of 21 patients with a normal alkaline phosphatase was 29 months and 9 months for 14 patients with elevated serum levels (P = 0.005). A similar observation was noticed for gamma-glutamyltransferase (P = 0.007). We concluded that mitoxantrone is not active in APUD tumours. Elevated alkaline phosphatase and gamma-glutamyltransferase are associated with a poor prognosis.


### Mitoxantrone in metastatic apudomas: a phase II study of the EORTC Gastro-Intestinal Cancer Cooperative Group

JP Neijt[1], AJ Lacave[2], TAW Splinter[3] BG Taal[4], CHN Veenhof[5], T Sahmoud[6] and CJM Lips[1]

[1]*Department of Internal Medicine, Utrecht University Hospital, Heidelberglaan 100, 3584 CX Utrecht, PO Box 85. 500, 3508 GA Utrecht, The Netherlands;* [2]*Hospital Gcneral de Asturias, Service Oncologia Medica, 33006-Oviedo, Spain;* [3]*Rotterdam University Hospital, Department of Medical Oncology, Dr. Molewaterplein 40, 3015 GD Rotterdam, The Netherlands;* [4]*Netherlands Cancer Institute, Antoni van Leeuwenhoekhuis, Department of Medical Oncology, Plesmanlaan 121, 1066 CX Amsterdam, The Netherlands;* [5]*Academic Medical Center, Department of Internal Medicine, Meibergdreef 9, 1105 AZ Amsterdam, The Netherlands;* [6]*European Organization for Research and Treatment of Cancer, Av. E. Mounier 83-Bte, 1200 Brussels, Belgium.*

**Summary** We performed a phase II study with mitoxantrone in patients with carcinoid tumours, islet cell tumours and medullary carcinomas of the thyroid. Thirty-five eligible patients received mitoxantrone 12 mg m$^{-2}$ i.v. every 3 weeks. Among 18 previously untreated patients, three responded (17%, 95% CI = 4–41%); no responses were achieved in 17 previously treated patients. Of the 21 patients who had carcinoid tumours, 11 were previously untreated and two achieved a response (18%, 95% CI = 2–52%). Overall response rate was 9% (95% CI = 2–23%). At a median follow-up of 43 months, median overall survival was 16 months. The median survival of 21 patients with a normal alkaline phosphatase was 29 months and 9 months for 14 patients with elevated serum levels ($P = 0.005$). A similar observation was noticed for γ-glutamyltransferase ($P = 0.007$). We concluded that mitoxantrone is not active in APUD tumours. Elevated alkaline phosphatase and γ-glutamyltransferase are associated with a poor prognosis.

**Keywords:** APUD tumours; mitoxantrone; prognostic factors; carcinoid tumours; islet cell tumours; medullary carcinomas of the thyroid

Carcinoid tumours, islet cell tumours and medullary carcinomas of the thyroid are considered to have common cell origin from the neural crest (Pearse, 1969). The three tumours share the ability to decarboxylate amines (APUD tumours). Aggressive surgical resection of the bulk of the disease is recommended. In patients with unresectable metastatic tumours, antihormonal measures and finally cytotoxic chemotherapy may improve symptoms. Because of the similar properties of these cell types, advanced disease is often treated by common chemotherapy protocols (Kessinger *et al.*, 1983; Saltz *et al.*, 1993).

The use of single drugs or combination chemotherapy regimens result in no response to a 40% response rate at best. However, it is suggested by experienced investigators that patients should undergo cytotoxic treatment as soon as symptoms appear that cannot be controlled otherwise (Moertel, 1987). The best response rates for carcinoid tumours have been achieved with 5-fluorouracil, doxorubicin and streptozotocin (between 20 and 30%). Combination chemotherapy is not better than single drugs. The best treatment results for islet cell tumours have been achieved with a combination of streptozotocin plus doxorubicin (Moertel *et al.*, 1992). However, severe nausea and vomiting with this regimen compromised the patients' quality of life, and other combinations with better therapeutic effectiveness and better clinical tolerance are needed.

Mitoxantrone seemed worth testing in APUD tumours because of its low gastrointestinal toxicity and similarity to doxorubicin, which is known to be active in this context (Moertel *et al.*, 1982). We performed a phase II study of mitoxantrone to determine its activity in patients with APUD tumours.

### Patients and methods

Patients admitted to this non-randomised study had histologically proven islet cell carcinoma, carcinoid tumours or medullary carcinomas of the thyroid not amenable to surgery. The sample size calculation was based on the two-stage Gehan (1961) design, aiming to include 14 patients and then adding other patients for each response seen in the first stage. This guarantees that the probability of an active treatment (real response rate ⩾ 20%) exhibiting no responses in the first 14 patients (that is a false negative result) is 0.05 and allows the therapeutic effectiveness to be estimated with a standard error of 10%. Prior chemotherapy was allowed because at that time some members of the EORTC Gastro-Intestinal Cancer Cooperative Group felt that a trial with a known active drug should be done prior to treatment with a new drug. Only patients with measurable metastatic progressive disease were entered. Informed consent was obtained according to policies applied in the individual participating institutions. No pharmaceutical firm was involved in the study.

Patients were excluded if they were older than 75, had received prior treatment with doxorubicin or prior radiotherapy to the single indicator lesion or had previous or other cancer. None of the patients received concurrent Sandostatin. At the start of chemotherapy the haematological status had to be favourable (white blood cells more than $3.5 \times 10^9 l^{-1}$ or platelets more than $100 \times 10^9 l^{-1}$), bilirubin less than 25 $\mu mol\, l^{-1}$, creatinine clearance more than 60 ml min$^{-1}$. The extent of measurable disease was assessed before treatment and after three and six treatment cycles.

Patients received mitoxantrone in a dose of 12 mg m$^{-2}$ i.v. over 30 min (in 100 ml of 5% dextrose solution) repeated every 3 weeks. Concurrent administration of a standard antiemetic was permitted. Drug administration was postponed for up to 3 weeks in the absence of full haematological recovery from the previous course (leucocytes more than 30000 $\mu l^{-1}$ and platelets more than 100 000 $\mu l^{-1}$). If full haematological recovery had still not occurred, dose adjustments were made according, to the lowest value of the leucocyte and platelet counts determined on day 15. Patients received at least three courses to be evaluable unless it was not in their best interest. Response was assessed using standard WHO (1979) criteria and evaluated after three cycles of treatment, unless severe toxicity or disease progression supervened and it was deemed to be against the patient's best

Correspondence: JP Neijt
Received 21 February 1994; revised 26 August 1994; accepted 30 August 1994

interests to continue. Early progression after one cycle was evaluated as such.

### Results

Between April 1985 and March 1990, 37 patients were enrolled in the study. Two patients were ineligible, one patient with no information after registration and another because of inadequate histology (follicular thyroid carcinoma). The present analysis is based on 35 eligible patients. Table I presents patient and tumour characteristics at entry. Median age at entry was 58.6 years (range 21.9–75). Time from diagnosis varied between 2 days and 12 years with a median of 19 months.

Mitoxantrone starting dosage (mg) varied between 16 and 25 (median 20). Number of treatment cycles varied between 1 and 15 (median 6). Eight patients had a dose reduction defined as a dose less than 90% of the first dose during at least one cycle (reductions for treatment-related toxicity). The total dose of mitoxantrone (mg) varied between 18 and 314 (mean $\pm$ s.d. = 117 $\pm$ 77, median = 108). When corrected for the surface area, the dose of mitoxantrone (mg $m^{-2}$) varied between 12 and 177 (median 61).

Side-effects of mitoxantrone were mild and included nausea and vomiting (grade 2, 23%; grade 3, 3%), stomatitis grade 1 (1 patient) and haemorrhage grade 2 and 4 occurred once. Other registered side-effects were infection, grade 2 (two patients), hair loss grade 1 (six patients) grade 2 (3 patients), leucopenia grade 3 in nine patients (26%) and grade 4 in two (6%).

**Table I** Patient and tumour characteristics at entry (%)

| | | |
|---|---|---|
| Sex | | |
| Males | 21 | (60) |
| Females | 14 | (40) |
| Tumour type | | |
| Islet cell tumours | 9 | (26) |
| Carcinoid tumours | 21 | (60) |
| Medullary carcinomas of the thyroid | 5 | (14) |
| Performance status (WHO) | | |
| 0–1 | 27 | (77) |
| 2–3 | 8 | (23) |
| Symptoms of endocrine hyperfunction | 18 | (51) |
| Prior surgery | | |
| No | 11 | (31) |
| Yes curative | 10 | (29) |
| Yes palliative | 14 | (40) |
| Prior radiotherapy | 3 | (9) |
| Prior chemotherapy | 17 | (49) |
| Prior hormone therapy | 5 | (14) |
| γGT, WHO grade[1] | | |
| 0 | 17 | (49) |
| 1 | 7 | (20) |
| 2 | 7 | (20) |
| 4 | 3 | (9) |
| Unknown | 1 | (3) |

[1]0, $\leqslant$ 1.25 N; 1, 1.26–2.5 N; 2, 2.6–5.0 N; 3, 5.1–10.0 N; 4, $\geqslant$ 10 N (N, normal value).

Table II presents the response data. Three patients (9%, 95% CI = 2–23%) achieved a partial remission. None of the responders had prior radiotherapy or chemotherapy. In the 18 previously untreated patients the response rate was 17% (95% CI = 4–41%). Duration of response was 8.5, 13.8 and 16.6 months. Two out of eleven patients with untreated carcinoid tumours responded (18%, 95% CI = 2–52%). Twenty-six patients died; 22 died from their malignancy, whereas the case of death was unknown for the other four patients. At a median follow-up of 43 months, the median survival of all patients was 16 months. No differences in survival could be detected between males and females, performance status at entry, the present of symptoms of endocrine hyperfunction, prior surgery, histological tumour type or levels of aspartate aminotransferase (ASAT) or alanine aminotransferase (ALAT). However, the survival of patients with an elevated alkaline phosphatase (WHO 1–4) was decreased compared with patients with a normal alkaline phosphatase. The median survival of 21 patients with a normal alkaline phosphatase was 29 months and for 14 patients with elevated serum levels it was 9 months ($P = 0.005$). A similar observation was noticed for γ-glutamyltransferase. Seventeen patients was normal values survived for 30 months, whereas those with abnormal values died after a median of 10 months ($P = 0.007$) (see Figure 1). No association was found between the presence of liver metastases and an elevation of these enzymes.

### Discussion

Treatment with mitoxantrone resulted in disease stabilisation in 43% of the patients. This is not an uncommon result in this usually slow progressive type of disease. Unfortunately, partial remissions were rare (9%), and it must be concluded that mitoxantrone has no important efficacy as a single drug in patients with APUD tumours. The three responses were achieved in the group of 18 non-pretreated patients, two of which occurred in 11 untreated carcinoid tumours (18%), but

**Figure 1** Duration of survival of patients with a normal γ-glutamyltransferase (γ-GT, WHO 0) or with an elevated γ-glutamyltransferase (γ-GT, WHO 1–4).

**Table II** Response to treatment according to tumour type ($n = 35$)

| | *Islet cell tumours* | *Carcinoid tumours* | *Medullary carcinomas of the thyroid* | *Total no. of patients (%)* |
|---|---|---|---|---|
| Evaluable patients | 9 | 21 | 5 | 35 (100) |
| Partial remission | | 2 | 1 | 3 (9) |
| No change | 6 | 7 | 2 | 15 (43) |
| Progression | 3 | 11 | 1 | 15 (43) |
| Early progression | | | | 1 (3) |
| Insufficient data | | 1 | | 1 (3) |

this response rate was judged to be to low to warrant further study. In untreated patients combination chemotherapy with doxorubicin and streptozocin results in response rates of about 60% (Moertel *et al.*, 1992). The results prove that chemotherapy can result in tumour shrinkage and that further studies are needed to identify new active drugs. The role of biologicals such as interferon-α (IFN-α) with or without cytotoxic drugs has to be further established (Oberg and Eriksson, 1991) and new drugs such as paclitaxel (Taxol), and 2′,2′-difluorodeoxicytidine (Gemcitabine) should undergo phase II testing. Because an established standard treatment is not available, such a study should preferably be performed in previously untreated patients aiming at a response rate above 30%.

Prognostic factors that determine outcome in APUD tumours suitable for chemotherapy are not well known. This study revealed liver function disturbance as measured by alkaline phosphatase or γ-glutamyltransferase to predict an unfavourable prognostic outcome in terms of survival. Future studies should report these patient characteristics as survival data are used as an end point of treatment efficacy.

### References

GEHAN EA. (1961). The determination of the number of patients required in a preliminary and follow-up trial of a new therapeutic agent. *J. Chron. Dis.*, **13**, 346–353.

KESSINGER A, FOLLEY JF AND LEMON HM. (1983). Therapy of malignant APUD cell tumours. *Cancer*, **51**, 790–794.

MOERTEL CG. (1987). An odyssea in the land of small tumors. *J. Clin. Oncol.*, **5**, 1502.

MOERTEL CG, LAVIN PT AND HAHN RG. (1982). Phase II trial of doxorubicin therapy for advanced islet cell carcinoma. *Cancer Treat. Rep.*, **66**, 1567–1969.

MOERTEL CG, LEFKOPOULO M, LIPSITZ S, HAHN RG AND KLAASSEN D. (1992). Streptozocin–doxorubicin, streptozocin–fluorouracil, or chlorozotocin in the treatment of advanced islet-cell carcinoma. *N. Engl. J. Med.*, **326**, 519–523.

OBERG K AND ERIKSSON B. (1991). The role of interferons in the management of carcinoid tumours. *Acta Oncol.*, **30**, 519–522.

PEARSE AG. (1969). The cytochemistry and ultrastructure of polypeptide hormone-producing cells of the APUD series and the embryologic, physiologic and pathologic implications of the concept. *J. Histochem. Cytochem.*, **17**, 303–313.

SALTZ L, LAUWERS G, WISEBERG J AND KELSEN D. (1993). A phase II trial of carboplatin in patients with advanced APUD tumors. *Cancer*, **72**(2), 619–622.

WHO. (1979). Handbook for Reporting Results of Cancer Treatment. World Health Organization: Geneva.